# Metallothionein – Immunohistochemical Cancer Biomarker: A Meta-Analysis

**DOI:** 10.1371/journal.pone.0085346

**Published:** 2014-01-08

**Authors:** Jaromir Gumulec, Martina Raudenska, Vojtech Adam, Rene Kizek, Michal Masarik

**Affiliations:** 1 Department of Pathological Physiology, Masaryk University, Brno, Czech Republic; 2 Central European Institute of Technology, Brno University of Technology, Brno, Czech Republic; 3 Department of Chemistry and Biochemistry, Mendel University in Brno, Brno, Czech Republic; Istituto dei tumori Fondazione Pascale, Italy

## Abstract

Metallothionein (MT) has been extensively investigated as a molecular marker of various types of cancer. In spite of the fact that numerous reviews have been published in this field, no meta-analytical approach has been performed. Therefore, results of to-date immunohistochemistry-based studies were summarized using meta-analysis in this review.

Web of science, PubMed, Embase and CENTRAL databases were searched (up to April 30, 2013) and the eligibility of individual studies and heterogeneity among the studies was assessed. Random and fixed effects model meta-analysis was employed depending on the heterogeneity, and publication bias was evaluated using funnel plots and Egger's tests.

A total of 77 studies were included with 8,015 tissue samples (4,631 cases and 3,384 controls). A significantly positive association between MT staining and tumors (vs. healthy tissues) was observed in head and neck (odds ratio, OR 9.95; 95% CI 5.82–17.03) and ovarian tumors (OR 7.83; 1.09–56.29), and a negative association was ascertained in liver tumors (OR 0.10; 0.03–0.30). No significant associations were identified in breast, colorectal, prostate, thyroid, stomach, bladder, kidney, gallbladder, and uterine cancers and in melanoma. While no associations were identified between MT and tumor staging, a positive association was identified with the tumor grade (OR 1.58; 1.08–2.30). In particular, strong associations were observed in breast, ovarian, uterine and prostate cancers. Borderline significant association of metastatic status and MT staining were determined (OR 1.59; 1.03–2.46), particularly in esophageal cancer. Additionally, a significant association between the patient prognosis and MT staining was also demonstrated (hazard ratio 2.04; 1.47–2.81). However, a high degree of inconsistence was observed in several tumor types, including colorectal, kidney and prostate cancer.

Despite the ambiguity in some tumor types, conclusive results are provided in the tumors of head and neck, ovary and liver and in relation to the tumor grade and patient survival.

## Introduction

Metallothioneins (MTs) are cysteine-rich low-molecular-mass intracellular proteins occurring in a wide variety of eukaryotes and constituting the major fraction of intracellular protein thiols [1]. The MT gene family consists of four subfamilies designated as MT-1 through to MT-4 in mammals. MTs are involved in many physiological and pathophysiological processes such as intracellular storage, transport and metabolism of metal ions, whereas they regulate essential trace metal homeostasis and play a protective role in heavy metal detoxification reactions [2], [3]. They can protect cells against UV/ionic radiation [4], [5] as well as cytotoxic alkylating agents including chemotherapeutics [6]–[9], modulate oxygen free radicals and nitric oxide, and inhibit apoptosis [10]–[12].

MTs are usually expressed at low levels, but they are inducible [13]–[16]. The synthesis of MT was shown to be increased during oxidative stress [17], [18] to protect the cells against cytotoxicity [19], [20], radiation and DNA damage [21]–[23]. Many studies have shown an increased expression of MT in human tumors of breast, colon, kidney, liver, lung, nasopharynx, ovary, prostate, salivary gland, testes, thyroid and urinary bladder [14]. MT expression in tumor tissues is mainly correlated with the proliferative capacity of tumor cells [24]. However, there are few exceptional cases, e.g. down-regulation of MT in hepatocellular carcinoma [25]. Nevertheless, these case-control and cohort studies give us inconsistent results regarding the association of MTs and tumor histology, staging, grading, prognosis, and survival. Although there is a number of good systematic reviews [2], [3], [11], [13], [14], [26]–[28], [29,] with particular interest in breast tumors [30], [31], a meta-analytic approach has not been employed yet. Thus, the aim of this study is to evaluate the associations between immunohistochemical MT staining and clinicopathological conditions, tumor type, stage, grade, prognosis, and survival using the meta-analysis.

## Materials and Methods

### Literature search

Search was performed in Web of science (Science citation index expanded 1945 to April 2013), PubMed (Medline 1968 to April 2013) search engines and in bibliographies of cited references. The following keywords were used: histo* OR immunohisto* OR IHC; metallothionein; cancer OR tumor OR tumour OR neoplas*; melanoma. The date of publishing and language were not restricting

### Selection criteria

Case-control and cohort studies regarding the associations between malignant neoplasms and metallothionein immunohistochemical staining were searched. Full text articles were included only. Following information were extracted from the studies: (1) MT level in malignant tumors and healthy/benign tissues, (2) MT level regarding the tumor stage, (3) tumor grade, (4) age and sex of patients, and (5) MT level and survival. The following data formats were accepted: (1) means, standard deviation and sample size, (2) sample size, means, P values and type of statistical test type (one- or two-tailed), and (3) sample size, P values, statistical test type and effect direction for continuous data and (1) odds ratios and 95% confidence intervals (CI), (2) 2×2 tables, and (3) Chi-squared and effect directions for dichotomous data. Continuous and dichotomous outcomes were combined. Cox proportional hazard model was used for survival meta-analysis. Univariate model of overall survival was used, hazard ratio and 95% CI was extracted from the studies. Studies with the sample size <6 participants and without histological verification of tumor were not included. If similar data was found in more than one study, studies with more extensive data set were used for the analysis. The eligibility of the studies for meta-analysis was evaluated by two authors (J.G. and M.R.).

### Coding of categorical variables

Since different scales regarding MT IHC staining were used across the studies, the following rules were applied: (1) when MT staining was encoded as positive/negative, no change was applied; (2) when percentage data was included, staining >10% was considered positive and vice versa; (3) when no percentage data was identified and data were encoded by 0–2 or 0–3 points, 0–1 was considered negative. Because >2 categories are used in grading/staging scales, Grades 2–3 were grouped and compared with Grade 1; stages 3–4 and 1–2 were grouped in a similar way.

### Statistical analysis

Odds ratios with 95% intervals were used as point estimates except for the survival analysis. For the survival analysis, hazard ratios with 95% confidence intervals were used. To assess heterogeneity across the studies, Higgins I^2^, describing the percentage of variability in point estimates was calculated [32]. The random effects model meta-analysis using the DerSimonian and Laird method was employed when a distinct heterogeneity was observed [33] (I^2^ more than 50.0%), otherwise, a fixed model was used. The model selection is based on a study by Borenstein et al. [34]. In that study, key assumptions of each model and mathematical bases are explained, and differences between the models are outlined; therefore, they will not be discussed in this paper. Subgroups were combined using the fixed effects. Within-subgroup estimates of tau-squared were not pooled. When the number of studies within the groups exceeded 4, the publication bias was evaluated using funnel plots and two-sided Egger's tests. Funnel plots of subgroups whose Egger's test p<0.05, are asymmetric. Comprehensive Meta-analysis Version 2 software (Biostat, Englewood, NJ) was used for the analysis.

## Results and Discussion

### Identification and characteristics of relevant studies

**Figure 1:**
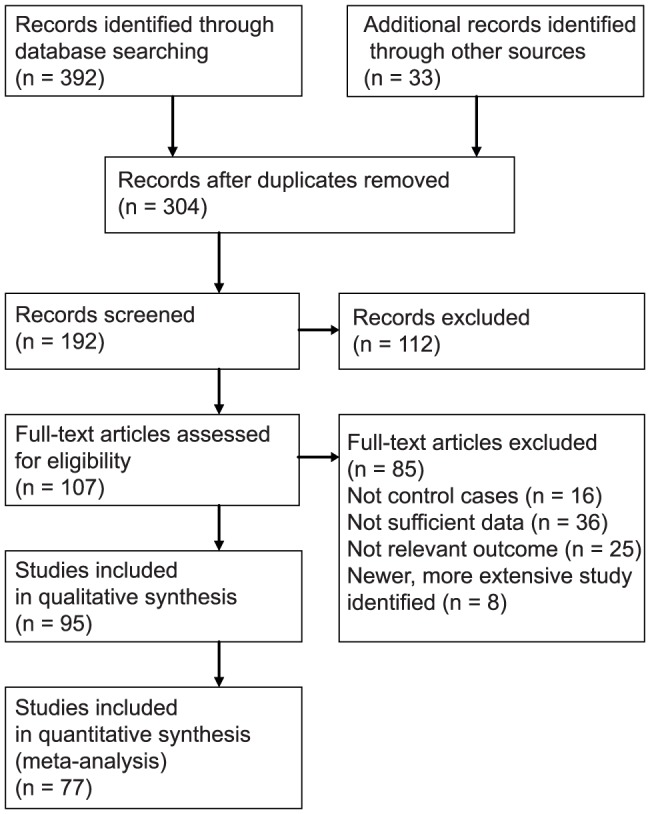
Flow chart showing the number of citations retrieved by database searching.

The acquisition process of studies is depicted in Fig. 1. A total of 77 articles were included in the final analysis after eliminating articles unsatisfying the selection criteria and duplicates. The set of 77 studies includes 8,015 tissue samples (4,631 cases and 3,384 controls). On average 95.2 patients were included per study, 1270 cases and controls were included in the largest study [35] and 12 cases and controls were included in the smallest study [36]. The date of publication ranged from 1987–2013 with the median of year 2002. In total, 51 North American and European studies, 20 Asian, 2 South American studies and one African study were included.

### MT isoform characteristics

Monoclonal antibody clone E9 was used in a majority of studies; this antibody shows affinity for both MT-1 and 2 isoforms. Anti-MT3 antibody was only used by Sens et al. in bladder cancer [37]. Strong positive associations of MT3 staining with the tumor grade were determined. Therefore, with the exception of Sens et al., all following analyzes concern the MT-1 and 2 isoforms.

### MT and patient characteristics

#### Patient age

First, the association of MT immunoreactivity and patient's age was analyzed. No significant association was determined using the fixed effects model meta-analysis (odds ratio, OR = 1.07; 95% confidence interval, CI, 0.48 to 0.628). No publication bias was identified (Egger's 2-tailed test p = 0.12). In total, 9 studies with 1,069 patients were included [25], [38]–[45]. The following tumors were included: bladder, breast, colorectal, hepatocellular, head and neck, and stomach.

This is a generally expected finding, no correlation of MT immunoreactivity and age was already reported in breast cancer patients [46]-[49]. No such age-dependent association is beneficial from the perspective of potential diagnostic use.

#### Patient gender

Consequently, the role of gender was analyzed. No publication bias was observed (p = 0.61) and no significant trend was detected using the fixed effect meta-analysis (OR = 0.99; 95% CI, 0.74 to 1.34). The analysis was performed on 9 studies reporting gender (944 patients). The following tumor types were included: bladder [44], colorectal [38], [39], [50], head and neck [41], [51], hepatocellular [25], and stomach [43]. Similarly as in the patients' age, no significance was expected. Such gender-independence is advantageous for appropriate tumor biomarker.

### Metallothionein as cancer biomarker

**Figure 2:**
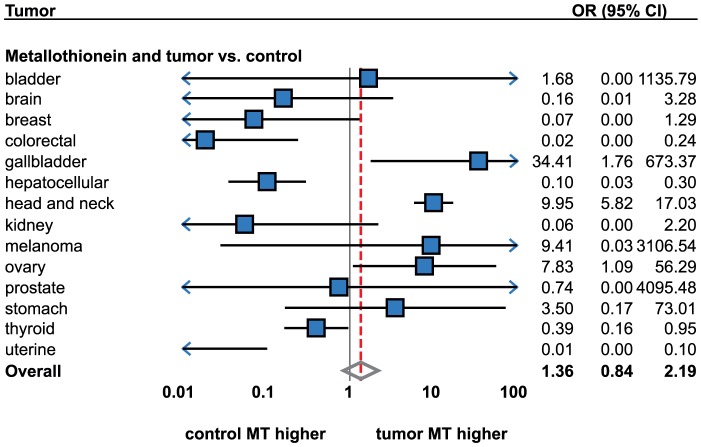
Forrest plot showing associations between metallothionein staining and tumors (tumors versus healthy controls). The result of meta-analysis for particular tumor types displayed instead of individual studies. For more detailed results see Table 1 and 2. Sorted alphabetically by tumor types. Forrest plot displayed as odds ratio and 95% confidence intervals. Red dashed line indicates the result for all tumor types together. OR, odds ratio; CI, confidence interval.

Consequently, the association of MT staining and tumor presence was analyzed. A total number of 31 studies were included (2,454 cases and healthy individuals). A high degree of heterogeneity was identified (Higgins I^2^ = 89.6%) when the meta-analysis was performed on all tumor types together (Fig. 2, Table 1). Therefore, no significant association between MT staining and tumor presence was identified using the random effects model (OR = 1.19; 95% CI, 0.47 to 3.01). No publication bias was identified. The high degree of heterogeneity between the studies calls for explanation. Therefore, a subgroup analysis by tumor types was performed.

**Table 1:** Association of MT staining and clinicopatological factors. Tumor type not taken into account.

Factor	Number of studies	Number of participants	OR/HR* (95% CI)	Heterogeneity		Publication bias	Model
				P-value	I^2^ (%)	P-value	
Age	9	1069	1.07 (0.82–1.39)	0.679	0.00	0.117	Fixed effects
Gender	9	944	0.99 (0.74–1.34)	0.393	5.08	0.611	Fixed effects
Tumor vs. control	31	2454	1.19 (0.47–3.01)	0.000	89.58	0.236	Random effects
Tumor stage	19	1237	1.15 (0.74–1.77)	0.000	60.83	0.987	Random effects
Tumor size	6	1962	1.37 (0.45–4.13)	0.000	90.88	0.194	Random effects
Tumor grade	33	2504	1.58 (1.08–2.30)	0.000	66.57	0.886	Random effects
Metastases, nodal + distant	21	1987	1.59 (1.03–2.46)	0.000	71.68	0.039	Random effects
Metastases, distant	6	741	1.56 (0.56–4.37)	0.014	64.82	0.075	Random effects
Metastases, nodal	15	1246	1.62 (0.98–2.68)	0.000	72.92	0.201	Random effects
Overall survival	10	2041	2.04 (1.47–2.81)	0.000	97.69	0.572	Random effects

Heterogeneity of studies analyzed using Cochran's Q-test (p-value displayed) and using I^2^. Egger's two-tailed test used for publication bias analysis (p-value displayed). * effect measure is odds ratio, OR, except for survival analysis using hazard ratio, HR. CI, confidence interval

#### Head and neck cancer

Head and neck tumors were analyzed most extensively, five studies were identified (Table 2). Tumors in the following locations were included: oral cavity [41], tongue [52], pharynx [53], and larynx [54], [55]. Except for Sundelin et al., all studies showed a significant increase of MT levels in tumorous tissues (OR = 9.95; 95% CI, 5.82 to 17.03). The fixed effects model meta-analysis was used due to low heterogeneity, I^2^ = 34.5. This is in agreement with our study based on voltammetric MT detection [56].

**Table 2:** Association of MT staining and clinicopathological factors – individual tumor types taken into account.

Tumor	Factor	Number of studies	OR/HR* (95% CI)	Heterogeneity		Model
				P-value	I^2^ (%)	
bladder	Tumor vs. control	2	1.68 (0–1135.79)	0.002	89.89	Random effects
	Tumor stage	3	1.56 (0.77–3.16)	0.354	3.64	Fixed effects
	Tumor grade	3	0.96 (0.21–4.33)	0.027	72.24	Random effects
	Metastases, nodal + distant	3	1.78 (0.57–5.51)	0.070	62.44	Random effects
	Metastases, nodal	2	1.96 (0.40–9.66)	0.022	80.89	Random effects
	Metastases, distant	1	1.31 (0.21–8.27)	-	-	-
	Overall survival	1	2.06 (1.26–3.37)	-	-	-
breast	Tumor vs. control	1	0.07 (0.00–1.29)	-	-	-
	Tumor stage	1	1.20 (0.59–2.43)	-	-	-
	Tumor size	1	1.73 (0.68–4.40)	-	-	-
	Tumor grade	5	1.85 (1.22–2.82)	0.096	49.19	Fixed effects
	Metastases, nodal + distant	3	2.57 (0.59–11.26)	0.001	86.55	Random effects
	Metastases, nodal	3	2.57 (0.59–11.26)	0.001	86.55	Random effects
colorectal	Tumor vs. control	1	0.02 (0.00–0.24)	-	-	-
	Tumor stage	5	1.27 (0.51–3.16)	0.000	83.09	Random effects
	Tumor size	2	0.71 (0.32–1.56)	0.299	7.15	Fixed effects
	Tumor grade	6	2.32 (0.86–6.29)	0.000	77.64	Random effects
	Metastases, nodal + distant	6	1.11 (0.38–3.24)	0.001	75.84	Random effects
	Metastases, nodal	4	1.12 (0.30–4.16)	0.001	82.13	Random effects
	Metastases, distant	2	1.04 (0.07–15.17)	0.050	73.87	Random effects
	Overall survival	1	1.05 (1.00–1.10)	-	-	-
esophageal	Metastases, nodal + distant	2	2.89 (1.17–7.15)	0.464	0.00	Fixed effects
	Metastases, nodal	1	2.30 (0.77–6.85)			Fixed effects
	Metastases, distant	1	4.78 (0.94–24.33)	-	-	-
gallbladder	Tumor vs. control	1	34.41 (1.76–673.37)	-	-	-
	Tumor grade	1	2.25 (0.37–13.71)	-	-	-
hepatocellular	Tumor vs. control	3	0.10 (0.03–0.30)	0.548	0.00	Fixed effects
	Tumor stage	1	3.53 (0.36–34.18)			-
	Tumor size	2	1.04 (0.12–9.42)	0.023	80.53	Random effects
	Tumor grade	2	0.47 (0.28–0.80)	0.832	0.00	Fixed effects
	Metastases, nodal + distant	1	0.62 (0.39–0.98)	-	-	-
	Metastases, distant	1	0.62 (0.39–0.98)	-	-	-
head and neck	Tumor vs. control	5	9.95 (5.82–17.03)	0.191	34.56	Fixed effects
	Tumor stage	2	2.02 (0.36–11.27)	0.123	58.04	Random effects
	Tumor grade	4	0.72 (0.22–2.40)	0.091	53.68	Random effects
	Metastases, nodal + distant	2	3.49 (0.25–48.04)	0.036	77.24	Random effects
	Metastases, nodal	2	3.49 (0.25–48.04)	0.036	77.24	Random effects
	Overall survival	2	3.14 (1.61–6.15)	0.826	0.00	Fixed effects
kidney	Tumor vs. control	2	0.06 (0.00–2.20)	0.043	75.47	Random effects
	Tumor stage	2	0.38 (0.07–2.13)	0.086	66.00	Random effects
	Tumor grade	2	1.74 (0.66–4.59)	0.001	90.91	Fixed effects
lung	Tumor grade	1	0.92 (0.05–18.12)	-	-	-
	Overall survival	2	1.03 (0.63–1.67)	0.622	0.00	Fixed effects
lung, adenoca.	Tumor grade	1	5.77 (0.29–116.67)	-	-	-
lung, squamous cell	Tumor grade	1	0.15 (0.01–2.81)	-	-	-
melanoma	Tumor vs. control	2	9.41 (0.03–3106.54)	0.000	93.39	Random effects
	Tumor size	1	4.85 (3.51–6.70)	-	-	-
	Metastases, nodal + distant	2	2.47 (1.30–4.70)	0.336	0.00	Fixed effects
	Metastases, nodal	1	2.23 (1.14–4.39)	-	-	-
	Metastases, distant	1	6.63 (0.81–54.61)	-	-	-
	Overall survival	1	7.16 (4.71–10.89)	-	-	-
ovary	Tumor vs. control	4	7.83 (1.09–56.29)	0.003	78.82	Random effects
	Tumor grade	2	3.08 (1.38–6.88)	0.616	0.00	Fixed effects
	Overall survival	1	1.58 (1.01–2.47)	-	-	-
prostate	Tumor vs. control	2	0.74 (0–4095.48)	0.000	95.32	Random effects
	Tumor grade	4	2.09 (0.86–5.07)	0.365	5.64	Fixed effects
	Gleason Grade	2	1.40 (0.47–4.14)	0.238	28.06	Fixed effects
	Overall survival	1	1.86 (1.79–1.94)	-	-	-
stomach	Tumor vs. control	3	3.50 (0.17–73.01)	0.000	95.71	Random effects
	Tumor stage	1	0.73 (0.33-1.63)	-	-	-
	Tumor grade	1	1.45 (0.67–3.14)	-	-	-
	Metastases, nodal + distant	1	0.59 (0.27–1.28)	-	-	-
	Metastases, nodal	1	0.59 (0.27–1.28)	-	-	-
	Overall survival	1	4.23 (1.8–9.94)	-	-	-
testes	Tumor stage	2	0.33 (0.10–1.03)	0.301	6.54	Fixed effects
thyroid	Tumor vs. control	3	0.39 (0.16–0.95)	0.182	41.39	Fixed effects
thyroid, follicular	Tumor vs. control	3	2.27 (1.11–4.63)	0.212	35.59	Fixed effects
thyroid, medullar	Tumor vs. control	1	0.11 (0.03–0.39)	-	-	-
thyroid, papillary	Tumor vs. control	3	0.23 (0.02–2.85)	0.026	72.64	Random effects
uterine	Tumor vs. control	1	0.01 (0.00–0.10)	-	-	-
	Tumor stage	2	1.53 (0.61–3.86)	0.538	0.00	Fixed effects
	Tumor grade	2	2.81 (1.17–6.8)	0.569	0.00	Fixed effects
	Metastases, nodal + distant	1	1.01 (0.27–3.79)	-	-	-
	Metastases, nodal	1	1.01 (0.27–3.79)	-	-	-

Heterogeneity of studies analyzed using Cochran's Q-test (p-value displayed) and using I^2^. Egger's two-tailed test used for publication bias analysis (p-value displayed). * effect measure is odds ratio, OR, except for survival analysis using hazard ratio, HR. No heterogeneity test and no model indicated when 1 study per factor analyzed. CI, confidence interval.

#### Ovarian cancer

A total of four studies were identified [57]–[60]. All studies except for Tan et al. reported a significant increase [59]. Using the random effects meta-analysis, significantly higher MT staining was identified in tumors as compared with healthy tissues (OR = 7.83; 95% CI, 1.09 to 56.30). These results are in agreement with Murphy et al. using Hg-binding assay and with Germain et al. [61], [62].

#### Thyroid tumors

Three studies were identified [63]–[65]. The following histological types were analyzed: papillary, follicular and medullar. When the histological type was not considered as a unit of analysis, non-significant differences were determined when compared with non-malignant tissues by the random effects model meta-analysis.

The subgroup meta-analysis by histological types revealed the sources of heterogeneous results; significantly higher MT levels were determined in follicular cancer (OR = 2.27; 95% CI, 1.11 to 4.62) using the fixed effects, no change in papillary cancer and a significant decrease in medullar cancer (OR = 0.10; 95% CI, 0.03 to 0.39). However, medullar cancer was only dealt with in one study [64]. Apart from IHC technique, contradictory results were demonstrated by mRNA expression analysis, decreased MT expression was demonstrated in papillary cancer and no expression change was demonstrated in follicular cancer [66].

#### Prostate cancer

No significant associations between MT staining and tumor presence were determined using the random effects model. Results observed in this tumor were contradictory. While one study showed significantly lower MT in the tumorous tissue [67], the other identified a significantly increased MT level [68]. The study by Wei et al., however, used benign prostatic hyperplasia as a control instead of healthy tissue [67].

While the alteration of zinc-metallothionein metabolism is an early sign of prostate cancer progression [69], the benign tissue is not an adequate biological sample for such analyzes. In addition, a study based on radioimmunoanalysis revealed a non-significantly decreased MT level in the tumorous tissue [70]. Although prostate cancer is unique regarding the zinc and MT metabolism [71], [72], no conclusive findings were provided by this meta-analysis and more studies are therefore needed.

#### Hepatocellular cancer

Three studies were identified. Although lower MT levels in the tumorous tissue were reported in all studies [73]–[75], only Lu et al. reported a significant decrease [75]. Despite this fact, the fixed model analysis revealed significantly lower MT levels (OR = 0.10; 95% CI, 0.04 to 0.30). Nevertheless, Lu et al. used specifically the MT1F isoform antibody instead of nonselective MT1-2, which was used by a majority of study groups.

The decreased MT level in the hepatocellular tumor is a well-established finding. Apart from immunohistochemistry, decreased expression of MT1F [75], MT1G [76], [77] and MT1X [25] was determined. ELISA-based detection also showed a decrease [78]. No change in MT levels was determined in one HPLC-based study [79]

#### Stomach cancer

Contradictory results were evident in three identified studies. While two research groups reported increased levels in tumorous tissues [80], [81], the study by Tuccari et al. demonstrated a significant increase in MT levels [43]. While all groups used the E9 antibody clone and included both early and advanced tumors, these contradictory results call for a further explanation by another study. However, contradictory results were shown using other approaches. Apart from IHC analyses, inconclusive results are provided also in gene expression- and radioimmunoanalysis-based methods. Elevation of MT1, 2, and 3 mRNA was determined in an RT-PCR-based study [80] and decrease of MT1 and 2 protein was determined in a radioimmunoanalysis-based study [82].

#### Bladder cancer

Two studies regarding bladder cancer were identified [83], [84]. Using the random effects model, no significant difference was identified. Contradictory results of studies were observed, while a positive association was demonstrated by Zhou et al. and a negative trend was reported by Saika et al. This contradiction may perhaps be explained by the use of benign tissues as controls instead of healthy individuals by Zhou et al. In addition to MT1 and 2 isoforms, Sens et al. demonstrated MT3 to be significantly up-regulated in tumorous tissues, and suggested its use as a potential biomarker for bladder cancer [37].

#### Kidney cancer

Two studies were identified [83], [85]. Although both of them show a decrease, the level of significance is achieved in one study only [85]. As a result, no significance is observed using the random effects model meta-analysis. While both IHC studies used the E9 antibody clone, differential affinity to MT-1 and 2 is not expected. Additionally to IHC determination, down-regulated MT1A, 1F, 1H, and 1G and up-regulated MT2A expression were observed also in other studies [14], [86], [87]. On a protein level, decreased MT levels were determined in the HPLC-based study [88].

#### Melanoma

MT levels in melanoma tissues were analyzed in two studies [89], [90]. Of those, a significant increase is presented only by Zelger et al. [90] and so a non-significant change in MT levels is observed using the random effects model.

#### Other tumor types

As compared with the previous chapter, only one study per tumor type was identified for several tumor types. Therefore, no meta-analytical approach was used. This applied to the following tumor types: breast, colon and rectum, gallbladder and uterine corpus.

Compared to non-malignant tissues, significantly higher MT staining was identified in gallbladder cancer [91], a significant decrease in colorectal [92] and cervical [93] tumors and no associations were observed in ductal breast tumors [94] and glioblastomas [95]. Immunohistochemical-based studies of other tumors were not identified.

While El Sharkarvy described non-significant changes in breast cancer tissues, other studies regarding breast cancer described intensive staining in the ductal type, while small or no staining was observed in lobular and papillary cancer [14], [46], [96]. There are number of reviews regarding MT expression in tumorous tissues. Pedersen, for instance, points to the issue of discrepancy between the MT expression in various tumors and the lack of overall consensus regarding the precise role of MT in human neoplasms [26]. According to these researchers, the enhanced expression is associated with the rapid proliferation or regeneration of normal cells and even with the aggressiveness and drug resistance of neoplasms [26]. This applies to the following tumors: kidney, breast, lung, nasopharynx, salivary gland, ovary, testes, urinary bladder, leukemia, and non-Hodgkin's lymphoma [14], [27], [97]. However, this meta-analysis only shows an agreement in ovarian and nasopharyngeal cancer while the rest of tumors did not exhibit any alterations of MT levels. On the other hand, the decrease of MT expression is associated with the poor prognosis namely of prostatic, hepatic, thyroid, brain and testicular tumors [14], [25], . An agreement is found in hepatic cancer only by this analysis.

Apart from differences between the individual tumor types, contradictory results were found even within particular histological types. According to the meta-analysis, such contradictions were observed in prostate, melanoma, and stomach tumors. While the high MT levels are associated with rapid proliferation and drug resistance and the low levels are associated with poor prognosis, it is likely that the MT levels change during the tumor progression. It is known that the zinc and metallothionein metabolism is being altered during early stages of tumorigenesis in prostate cancer, [69], [103], [104]; a similar tendency is expected in other histological types. Thus, a careful selection of controls is crucial; benign controls are therefore not an optimal biological material to demonstrate changes in MT/zinc levels. Additionally, it was reported that healthy tissues adjacent to a tumor vary distinctly as compared with the healthy tissues (i.e. tissues of patients without tumors) regarding zinc and MT levels. Considering the fact that MT is tightly related to oxidative stress buffering [11], the increase in oxidative stress affects the MT expression. Therefore, radiation, cytostatic drug therapy, or long-term stress must be taken into account when evaluating the MT levels.

### Tumor stage

**Figure 3:**
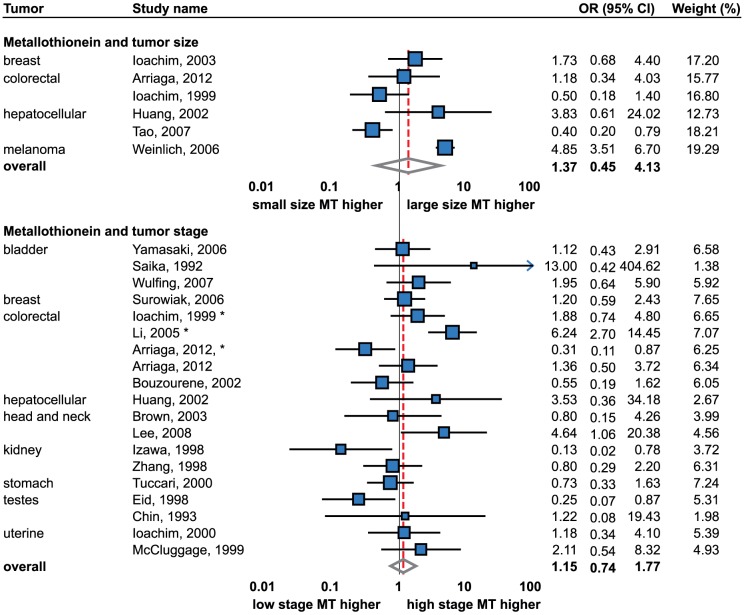
Forrest plot of studies reporting the association of metallothionein staining and tumor size and stage. Random effects model used for both outcomes, Relative weight of individual studies displayed in %. * indicates studies using Dukes staging system. For more detailed results see Table 1 and 2. Sorted alphabetically by tumor types. Forrest plot displayed as odds ratio and 95% confidence intervals. Red dashed line indicates the result for all studies together. OR, odds ratio; CI, confidence interval.

Although there are outlines that poor prognosis is associated with the higher MT expression in some tumors, for example breast tumors [30], [40], [49], no significant associations were determined by this meta-analysis (OR = 1.15; 95% CI, 0.74 to 1.77). No publication bias was observed (Egger's 2-tailed p = 0.99) and a vast majority of studies did not reveal any significant trends. In total, 1,237 samples were analyzed (Fig. 3).

Subsequently, the perspective of individual tumor types was taken into account. A colorectal cancer pT staging of two included studies did not reveal any significance using the meta-analysis [38], [105]; however, conflicting results were determined in colorectal cancer evaluating the Dukes staging. While a negative association (i.e. lower MT staining in higher stages) was revealed by one study [38], a positive association was determined by another study [106] and non-significant association was determined in another study [39]. Rather confusing was a study using radioimmunoanalysis with a positive association of MT staining and Dukes stage but no association to TNM staging [82].

Using the meta-analysis, no association of MT staining and tumor stage was observed in bladder [44], [83], [107], endometrial [108], [109], testicular [6], [98], kidney [110], [111], and head and neck cancer [41], [51]. There are also studies regarding tumors of breast [45], liver [74], and stomach [43] showing no significant association between MT staining and the tumor stage (Table 2). Nevertheless, each tumor was represented only by one study and no meta-analytical approach was applied. Therefore, more studies are needed.

### Tumor size

The association of MT staining and tumor size was a further subject of the meta-analysis. Six studies were included (1,962 samples); no association was observed using the random effects model and no publication bias was determined (Fig. 3).

When individual tumor types were evaluated separately, no significant trends were observed in colorectal cancer using the fixed effects and in hepatocellular cancer using the random effects model. Breast cancers and melanomas were identified in one study, with the only positive association found in melanoma. Additionally to IHC, no correlation of MT immunopositivity and tumor size was described by other researchers either [40], [46]–[49], [112]. Thus, according to our results and previous studies, MT staining is considered tumor size independent.

### Tumor grade

**Figure 4:**
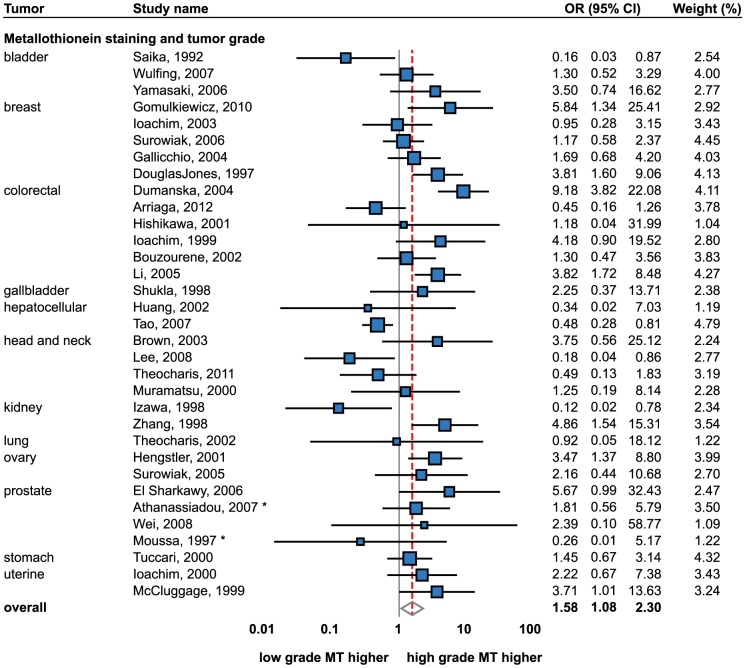
Forrest plot of studies reporting the association of metallothionein staining and tumor grading. Random effects model used. Relative weight of individual studies displayed in %. For more detailed results see Table 1 and 2. * indicates studies using Gleason grading. Sorted alphabetically by tumor types. Forrest plot displayed as odds ratio and 95% confidence intervals. Red dashed line indicates the result for all studies together. OR, odds ratio; CI, confidence interval.

The association of MT staining with the histological grade was studied most extensively; a total of 32 studies (2,504 samples) were identified (Fig. 4). Although a relatively high degree of heterogeneity between the studies was observed (I^2^ = 67.2%), still a significant positive association (i.e. higher MT in higher-grade tumors) was detected using the random effects model (OR = 1.61; 1.107–2.35). No publication bias was observed (p = p = 0.74).

Consequently, this association was analyzed in particular tumor types. Colorectal cancer was studied most with six studies being included [38], [39], [50], [105], [106], [113]. A positive trend was observed in only two studies [106], [113]. Thus, the random effects meta-analysis revealed no association between IHC staining of MT and the tumor grade.

Contrarily, a strong positive association was determined in breast tumors using the fixed effects model (OR = 1.85; 95% CI, 1.22–2.82). A total number of five studies of this tumor type were found [40], [45], [114]–[116] and through the established positive association, a significant trend was observed in two studies only [114], [116]. These IHC-based results are in agreement with findings based on other techniques, a similarly positive association was observed by numerous authors [46], [47], [49], [112], [117]–[119].

Four studies regarding head and neck tumors were included [41], [42], [51], [120]. However, a significant trend was observed only in one study [41]. Thus, no significant association was observed the using random effects model.

Prostate cancer is particularly interesting from the perspective of MT immunostaining. Four studies were included in total [67], [121]–[123]. Athanassiadou and Moussa used Gleason scale as a measure of tumor grading. When the grading scale was not taken into account, no trend was identified using the fixed effects model. However, the analysis suggests, that only Gleason scale shows no association; by contrast, the tumor grading was positively associated with MT staining (OR = 4.65; 1.01–21.52) using the fixed effects.

Using the fixed effects model, a positive association with the tumor grade was also determined in two studies of ovarian (OR = 3.08; 1.38–6.88) [124], [125] and two studies of endometrial cancers (OR = 2.82; 1.17–6.80). However, no MT-grading association was determined in ovarian cancer using Hg-binding assay [61].

On the other hand, a negative association of tumor staging was observed in hepatocellular cancer using the fixed effects, (OR = 0.43; 0.28–0.80). Thus, HCC is considered as the only histological type showing lower MT staining in higher tumor grades. However, only two studies were included and this is why the finding is still of a limited predictive value [25], [74].

Inconsistent results were observed in bladder and kidney cancers. While a non-significant association was identified in both tumor types, both positive [111] and negative [110] associations was determined in the case of renal cancer. In terms of bladder cancer, a negative association was demonstrated by Saika et al. [83], while non-significant trends were observed by other two studies [44], [107]. Additionally, a decreased MT3 gene expression was associated with higher-grade tumors [37].

Stomach [43], lung [126] and gallbladder [91] tumors were represented by only one study each, but non-significant associations between the tumor grade and MT staining were determined in all these studies. Lung tumors were studied by their histological type; however, non-significant associations were described either in adenocarcinomas or squamous cell carcinomas [126]. Other histological types were not included.

Taking into account the described relations of MT immunostaining with the increased proliferation and cytostatic resistance [14], [27], [97], it is not surprising that the associations of MT levels with tumor grading were studied to high extent. Meta-analysis results indicate a positive association. However, the results of this meta-analysis also indicate that the MT-grading associations are tumor specific. While a positive association was determined in most tumors, a negative trend was determined in hepatocellular cancer and inconsistent results were found in colorectal, bladder and kidney tumors. While a more or less distinct pattern is evident in all tumors, data are still lacking to prove whether the general positive association of MT staining and tumor grade may be generalized for these tumor types. While the low MT levels are associated with a worse prognosis and the high MT levels with increased proliferation [26], the MT levels vary during the disease progression. It is likely that the heterogeneity observed in some tumor types is a consequence of this phenomenon. Therefore, a precise understanding of MT level fluctuation in individual tumor types during the progression of disease is needed. Understanding the temporal changes will provide better comprehension of cellular tumor mechanisms and may predict a possible development of cytostatic resistance.

### Lymph node and metastatic status

**Figure 5:**
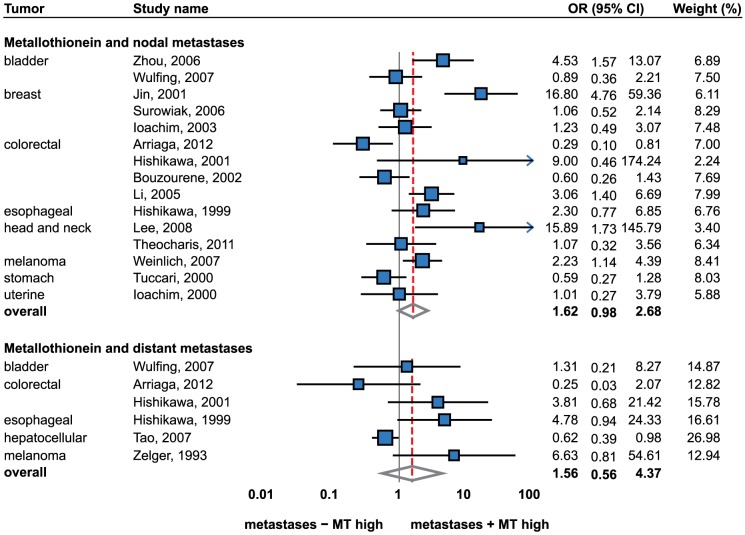
Forrest plot of studies reporting the association of metallothionein staining and nodal and distant metastases. Random effects model used for both outcomes, Relative weight of individual studies displayed in %. For more detailed results see Table 1 and 2. Sorted alphabetically by tumor types. Forrest plot displayed as odds ratio and 95% confidence intervals. Red dashed line indicates the result for all studies together. OR, odds ratio; CI, confidence interval.

Consequently, the association of the metastatic status and MT staining was taken as a subject of the meta-analysis. Included were 15 studies comparing nodal metastases and 6 studies comparing distant metastases (Fig. 5). Using the random effects model meta-analysis, a positive association of the metastatic status and MT staining was observed (OR = 1.59; 95% CI, 1.03–2.46). However, a high degree of heterogeneity between the studies (I^2^ = 71.68%) and publication bias were observed (p = 0.04). Nevertheless, when nodal and distant metastases were analyzed separately, no significant trends were observed either in nodal or distant metastases.

Consequently, individual tumor type was considered as a unit of the analysis. A significant association was observed in esophageal cancer (OR = 2.89; 1.17 to 7.15) [127], melanoma (OR = 2.47; 1.30 to 4.70) [90], [128] and hepatocellular cancer (OR = 0.62; 0.39 to 0.98) [25]. Notably, only one study per tumor type was included regarding esophageal and hepatocellular cancers.

Tumors of colon and rectum brought ambiguous results. While no association was identified by the meta-analysis, still one study demonstrated a negative association [38], two studies demonstrated a positive association [50], [106] and one study did not reveal a significant trend [105]. The remaining tumor types, including bladder [84], [107], breast [40], [45], [119], head and neck [41], [42], stomach [43], and uterine cancers [108] indicated no significant association between MT immunostaining and the presence of tumor metastases. However, it must be taken into account that the power of the meta-analysis for individual tumor types is limited due to the limited number of studies. Hence, by combining the tumor types the power of the analysis increases and the combined result indicates a significant positive association. However, there is still a lack of IHC data to make conclusive remarks on all tumor types.

Apart from the immunohistochemical analysis, no correlation between the lymph node status and the metastatic potential was described in breast cancer by several investigators [46]–[49], [112]. A significant association between the lymph node status and metastases was observed in one study only [129]. Schmid et al. suggest a higher probability of MT-positive tumors to develop metastases [130]. With regard to non-small cell lung cancer, no difference in the expression of all functional MT-1 and 2 isoforms was determined in another study [131].

### Survival analysis

The association of MT immunostaining and patient prognosis was evaluated by several researchers. However, various approaches were used. Univariate Cox proportional hazard model was included in the analysis. Unless noted otherwise, overall survival was used. Additionally, odds ratios, which reported the associations of MT staining with cancer-specific deaths, were also included. Median survival-based data were not evaluable by the meta-analysis and thus were not included.

**Figure 6:**
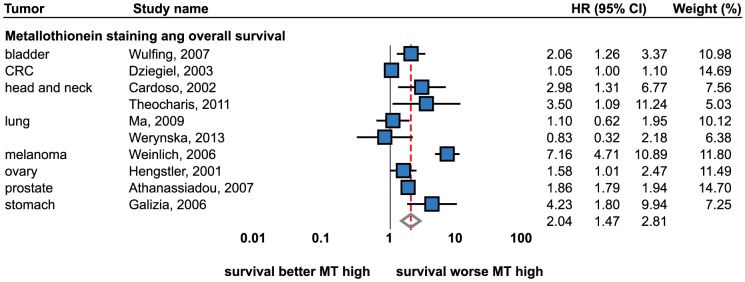
Forrest plot of studies reporting the association of metallothionein staining and the overall survival. Analysis based on Cox-proportional hazard model. Random effects model used. Relative weight of individual studies displayed in %. For more detailed results see Table 1 and 2. Sorted alphabetically by tumor types. Forrest plot displayed as hazard ratio and 95% confidence intervals. Red dashed line indicates the result for all studies together. HR, hazard ratio; CI, confidence interval.

Firstly, studies describing Cox proportional hazard model were analyzed. A total of 10 studies were included dealing with bladder, colorectal, head and neck, lung, melanoma, ovarian, prostate and stomach tumors (Fig. 6). A significant positive association (i.e. up-regulated MT associated with a worse prognosis) was determined using the random effects model (HR = 2.04; 95% CI, 1.47 to 2.81). No publication bias was determined (p = 0.57).

The significant positive association was determined in head and neck cancers (HR = 3.14; 95% CI, 1.61 to 6.15, fixed effects) [42], [132] while no association was determined in lung tumors [131], [133]. Both small cell and non-small cell tumors were included. Other tumor types were included in one study. The significant positive association was determined in the cancers of stomach [81], head and neck [42], prostate [121], bladder [107], head and neck [132], ovary [124], colon and rectum [134], and melanoma [35]. No association was identified in kidney tumors [135]. In addition to IHC analyses, poorer prognosis was associated with the higher MT1F and MT2A gene expression in non-small cell lung cancer in a qRT-PCR based study [131] and with higher MT protein levels in colorectal cancer in a radioimmunoanalysis-based study [82].

In addition to Cox model, retrospective studies reporting the following survival-related outcomes were analyzed: cancer-specific death, disease-free survival, short-term survival. While different outcomes were analyzed, the meta-analytical approach was limited in these studies. High MT expression was associated with poorer survival (OR = 3.42; 1.06 to 11.04) in melanoma patients, while an inverse effect was identified in patients with colon and rectum tumors. In those patients, lower MT levels were associated with a worse prognosis (OR = 0.41; 0.18 to 0.95). Univariable analysis of cancer-specific death was a subject of four studies. The following tumor types were included: breast [45], esophagus [136], head and neck [51], and stomach [43]. Neither any of the studies nor the result of the meta-analysis showed an association between cancer-specific death and MT staining. According to Joseph et al., no association was observed between MT staining and short-term survival [137]. Disease-free survival was analyzed in one study [36], which demonstrated a positive association in gastrointestinal stromal tumors (OR = 6.12; 1.12 to 33.52) and no association in leiomyosarcomas.

## Conclusions

The associations of immunohistochemical MT staining and various clinicopathological conditions of patients with tumors were analyzed using the meta-analytical approach. To date, it is the first meta-analysis regarding MT in pathological conditions. This meta-analysis was conducted only in studies based on immunohistochemical detection due to indubitable advantages of this method, which clearly identifies tumorous tissues. Therefore, effects of adjacent tissues on gene expression measurements are eliminated. In addition, the largest number of studies was based on immunohistochemistry. Therefore, the IHC-based results are of great statistical power.

More intensive MT staining in tumorous tissues compared to healthy tissues was observed in head and neck and ovarian tumors and a negative association was determined in liver tumors. No significant associations were identified in breast, colorectal, prostate, thyroid, stomach, bladder, kidney, gallbladder, and uterine cancers and in melanoma. However, more studies are needed in tumors showing insignificant results to confirm or disprove finding that the MT level remains unchanged. Most “insignificant” tumors is often represented by only two studies, which are often conflicting.

While no associations were identified between MT and tumor staging, a positive association was identified with the tumor grade. In particular, strong associations were observed in breast, ovarian, uterine and prostate cancers. Conversely, a negative association between MT staining and hepatocellular tumors was determined in this analysis. Borderline significant association of metastatic status and MT staining was determined in all tumors, in esophageal cancer in particular. Significant association between patient's prognosis and MT staining was also demonstrated.

However, this study has several limitations. Despite the mentioned advantages of immunohistochemistry, the semi-quantitative analysis is to a certain extent always subjective. Moreover, antibodies show affinity to a broad spectrum of MT1 and 2 isoforms. Nevertheless, gene expression-based studies indicate that MT level alterations are confined to certain isoforms. Such differences between isoforms may cause between-study discrepancies, which was evident in several tumor types, including colorectal, kidney, and prostate cancers. Therefore, techniques capable to distinguish MT isoforms on the protein level are desirable, e.g. capillary-based electrophoresis [138]. Despite this fact, such heterogeneity might also be due to MT fluctuation during the development of cancers, high levels being associated with the high rate of proliferation and cytostatic resistance, and low levels being associated with poorer prognoses. Therefore, precise understanding of MT level fluctuation in individual tumor types during the progression of disease is needed. Understanding the temporal changes will provide better comprehension of cellular tumor mechanisms and may predict possible development of cytostatic resistance. Moreover, the association of MT staining was investigated by researchers in certain tumor types more than in others. Therefore, the overall results (i.e. without taking into account the specific type of tumor) are biased toward more published tumor types and a cautious interpretation of the overall results is therefore necessary.

Despite these drawbacks, conclusive results are provided in some tumor types and in relation to tumor grade and patient survival by this meta-analysis. It is however still necessary to clarify the ambiguity of the association between MT staining and colorectal, lung, kidney, and prostate tumors.

## Supporting Information

Checklist S1 **Prisma 2009 checklist.**
(DOC)

Figure S1 **Prisma 2009 flow diagram showing the number of citations retrieved by database searching.**
(DOC)
