# Supplementary material for: Metallothionein – Immunohistochemical Cancer Biomarker: A Meta-Analysis
Source: PLoS One. 2014 Jan 8;9(1):e85346. doi: 10.1371/journal.pone.0085346 (PMC3885711; doi:10.1371/journal.pone.0085346)
Supplement: Figure S1 — Prisma 2009 flow diagram showing the number of citations retrieved by database searching. (DOC) [file pone.0085346.s002.doc]

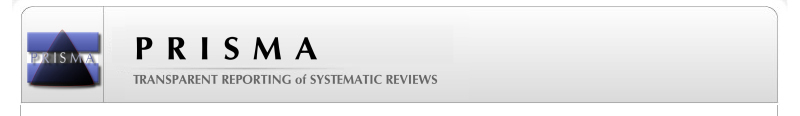
**PRISMA 2009 Flow Diagram**

**Screening**

**Included**

**Eligibility**

**Identification**

Records identified through database searching
(n = 392)

Additional records identified through other sources
(n = 33)

Records after duplicates removed
(n = 304)

Records screened
(n = 192)

Records excluded
(n = 112)

Full-text articles assessed for eligibility
(n = 107)

Full-text articles excluded, with reasons
(n = 85)

Studies included in qualitative synthesis
(n = 95)

Studies included in quantitative synthesis (meta-analysis)
(n = 77)
